# Volumetric stability of moldable octacalcium phosphate in guided bone regeneration: a CBCT-based ex vivo study

**DOI:** 10.1186/s40729-025-00631-9

**Published:** 2025-06-05

**Authors:** So-Ra Lee, Jooseong Kim, Woong Kim, Seok-Jun Kim, Yong-Gun Kim, Won-Pyo Lee

**Affiliations:** 1 Department of Periodontology, School of Dentistry, Chosun University, Gwangju, Republic of Korea; 2 School of Materials Science and Engineering, Yeungnam University, Gyeongsan, Republic of Korea; 3 Institute of Well-Aging Medicare & Chosun University G-LAMP Project group, Chosun University, Gwangju, Republic of Korea; 4 Department of Integrative Biological Sciences & BK21 FOUR Educational Research Group for Age-associated Disorder Control Technology, Chosun University, Gwangju, Republic of Korea; 5 Department of Biomedical Science, Chosun University, Gwangju, Republic of Korea; 6 Department of Periodontology, School of Dentistry, Kyungpook National University, Daegu, Republic of Korea

**Keywords:** Bone substitute, Guided bone regeneration, In vitro, Membrane, Octa calcium phosphate

## Abstract

**Purpose:**

The objective of this study was to evaluate the effect of flap suturing on the movement of graft materials during Guided Bone Regeneration (GBR) and to analyze the stability of moldable octacalcium phosphate (mOCP) depending on the type of graft material and membrane fixation method using Cone Beam Computed Tomography (CBCT).

**Methods:**

A total of 60 standardized rectangular-shaped bone defects were created in the alveolar ridges of mandibles from 5–6-month-old pigs (20 defects per group), and implants (4.0 mm in diameter, 10.0 mm in height) were placed into each defect. The control group employed particle-type OCP and a collagen membrane, experimental group 1 utilized particle-type OCP and a collagen membrane with fixation pins, and experimental group 2 employed mOCP and a collagen membrane. CBCT analysis was performed to evaluate changes in horizontal thickness (HT) at the grafted sites.

**Results:**

CBCT analysis revealed that the percentage reduction in HT at the implant shoulder level was significantly lower in experimental group 1 (16.7%) and experimental group 2 (16.3%) compared to the control group (31.5%), with no statistically significant difference observed between experimental groups 1 and 2.

**Conclusion:**

The use of mOCP in guided bone regeneration demonstrated comparable volumetric stability to grafts utilizing collagen membranes fixed with titanium pins, suggesting its potential to simplify surgical procedures by eliminating the need for additional fixation devices.

## Background

Following tooth loss, resorption of the alveolar bone is a common alteration to periodontal tissue. Typically, partial bone defects emerge in edentulous regions, necessitating guided bone regeneration (GBR) to facilitate alveolar bone reconstruction [1, 2]. GBR is the preferred method for augmenting localized defects in the alveolar bone. Currently, the combination of particulate xenogenic bone substitutes with collagen membranes (CM) represents the most widely used and well-documented approach for enhancing peri-implant defects [1, 2]. Numerous preclinical and clinical studies have demonstrated that effective bone integration and maintenance of alveolar bone volume can be achieved when GBR is applied to the exposed surfaces of implants. Furthermore, findings suggest that the survival rates for simultaneous GBR and implant placement are comparable to those of implants placed in intact alveolar bone without GBR [3, 4].

Autologous bone grafting remains the gold standard for reconstructing bone defects. However, it is constrained by the availability of bone volume and the necessity for additional surgical sites. In order to address these issues, several alternative materials have been developed and utilized, including allogeneic bone, xenogeneic bone, and synthetic bone. However, research continues to explore even better materials [5]. Synthetic bone has garnered increasing attention due to advantages such as mass production capability, cost-effectiveness, and a reduced risk of disease transmission [6, 7].

The bone graft material used in this study is a synthetic bone based on octacalcium phosphate (OCP). OCP is a synthetic bone graft material that has gained attention for its noteworthy capacity to facilitate osteoblast differentiation, which serves as a precursor to biological apatite crystals. It stimulates osteoblast activity while undergoing a transformation into hydroxyapatite (HA) within a physiological setting, thereby enhancing bone regeneration by incorporating HA produced through the hydrolysis of OCP [8–11]. The moldable OCP (mOCP) used in this study acquires a clay-like viscosity when exposed to water. This increased cohesion prevents the bone graft site from being washed away by fluids, allows tailoring to specific defect types, and improves overall stability [12]. While the concept of ‘sticky bone’ has been previously investigated using autologous materials such as platelet-rich fibrin (PRF) and concentrated growth factors (CGF), these approaches rely on patient-derived blood components that are inherently variable in composition and offer only transient biological activity [13, 14]. In contrast, the mOCP used in the present study is a fully synthetic formulation consisting of particulate octacalcium phosphate integrated with a hyaluronic acid-based hydrogel. Hyaluronic acid, a naturally occurring and highly biocompatible polysaccharide, serves as a viscoelastic carrier matrix that enhances the moldability and cohesiveness of the graft material while supporting cellular infiltration and promoting tissue regeneration. Furthermore, hydrogel systems such as those based on hyaluronic acid are widely recognized for their physicochemical resemblance to native tissues and their minimal interference with metabolic processes, making them suitable as carrier vehicles in regenerative applications [15]. Collectively, these attributes render the OCP–hyaluronic acid composite a clinically convenient and reproducible alternative to conventional autologous fibrin-based grafts. To the best of our knowledge, this study is the first to report the fabrication of a moldable synthetic bone graft by combining OCP with a hyaluronic acid hydrogel.

Resorbable collagen membranes are widely used as barrier membranes due to their ease of handling and low risk of disease transmission. However, resorbable collagen membranes have the disadvantage of being difficult to maintain in space due to their unfavorable mechanical properties in terms of resistance [16]. Therefore, if only particulate bone grafts and resorbable membranes are used, not only may microscopic movement of the membrane occur, but its resistance to displacement and collapse may be inadequate. The unstable mechanical properties of these absorbable membranes can result in membrane collapse and displacement of portions of the graft material during flap closure or under compressive forces at the augmentation site during the healing phase [17–19]. Although GBR using particle grafts and resorbable membranes is a viable method for alveolar bone reconstruction, the mechanical properties of the resorbable membrane may not be optimal to provide the required stability [16].

The occurrence of micro-movement between the defect site and the bone graft material has been demonstrated to impede the process of bone regeneration, with the potential to result in the formation of fibrous tissue, which is an unfavorable outcome for successful healing [20–22]. A substantial body of research has been conducted to document the efficacy of various membrane fixation techniques in promoting the success of GBR [23–26]. The use of fixation materials, such as pins, to enhance the stability of the membrane has been demonstrated to markedly reduce the movement of both the bone graft and the membrane, thereby improving the success of GBR procedures [23]. In particular, Mir-Mari et al. emphasized the necessity of utilizing fixation pin (FP) to anchor resorbable membranes in conjunction with particulate bone grafts [27]. As such, the stability of the volume following GBR is influenced by several key factors, including the intrinsic properties of the bone graft material, the manner in which membranes and pins are applied, and the manipulation of the flap [28–30].

Therefore, the aim of this study was to evaluate the physicochemical properties of mOCP—including crystallographic structure, surface morphology, and porosity—and to assess its volumetric stability in GBR using an ex vivo pig mandible model and CBCT-based dimensional analysis, with and without FP fixation, in order to determine its potential as a standalone, moldable grafting material.

## Materials and methods

### Materials

**Fig. 1:**
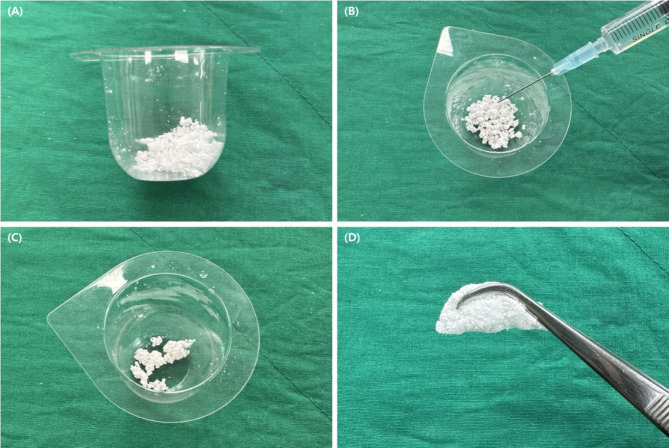
Workability of moldable octacalcium phosphate (mOCP). (**A**) The bowl containing mOCP after opening. (**B**) Hydration of mOCP in the provided bowl using sterile saline. (**C**) The mixture is evenly mixed using a spatula. (**D**) The preparation is completed in the form of a sticky grafting material

This study employed a comparative analysis of three distinct GBR methodologies. The control group employed a particulate OCP (pOCP) bone substitute (Bontree^®^; powder type, 0.3–0.85 mm; Hudens-Bio, Gwangju, Korea) in conjunction with a resorbable membrane (Remaix^®^; Matricel GmbH, Herzogenrath, Germany). Experimental group I comprised a pOCP bone substitute, a resorbable membrane, and FPs (BTS75-30; Osung, Gimpo, Korea). Experimental group II employed the use of a mOCP bone substitute in conjunction with a resorbable membrane. The production process has been previously described in detail [10]. To enhance the workability of the bone graft material and impart non-dispersive properties, mOCP was prepared (Fig. 1). Sodium hyaluronate (Bloomage Freda Biopharma Co., Ltd, China) was purchased and dissolved in deionized water to achieve a concentration of 5 mg/ml, with the solution pH adjusted to 7.0–8.0 using sodium hydroxide (NaOH). The resulting hydrogel was then uniformly mixed with pOCP. Finally, mOCP was obtained through lyophilization (Ilshin Biobase Co., Ltd, Korea). Samples were stored under cool and dry conditions.

### Characterization of mOCP

The phase compositions and microstructures of the samples were examined using a range of analytical techniques. Phase changes were investigated via X-ray diffraction (XRD; X’Pert PRO MPD, Malvern Panalytical, Malvern, UK) in the range of 3° to 60°, with intervals of 0.02°. The Joint Committee on Powder Diffraction Standards (JCPDS) file was utilized to identify the peak positions of the main phases. The morphologies of the prepared samples were analyzed using field-emission scanning electron microscopy (FE-SEM; S-4700, Hitachi, Tokyo, Japan). Prior to testing, the samples were sputter-coated with a 25-nm-thick platinum layer to enhance electrical conductivity, as this is a standard procedure in this field of study. Porosity measurements were conducted using a porosimeter (AutoPore 9620, Micromeritics Co., USA), which applied external pressure to forcibly intrude mercury into the pores of the porous samples. The amount of mercury intruded was calculated based on the applied pressure to determine the porosity. The porosity of the mOCP was evaluated through the measurement of the dimensions and mass of a parallelepiped material with a volume of 2 cm³. The porosity of the bone graft material was determined using the Washburn equation (D = -4γcosθ/p), which leverages the non-wetting properties of mercury for nearly all substances. By applying pressure to the penetrometer, it was observed that mercury infiltrated the pores, resulting in a decrease in the capillary height of the mercury. This was measured as a function of pressure. The reduction permitted an analysis of porosity based on the volume of mercury that was able to penetrate the material, in accordance with the principles of mercury porosimetry. The elemental composition of mOCP was characterized using transmission electron microscopy-energy dispersive spectrometry (TEM-EDS). The specimen for TEM-EDS analysis was prepared by grinding mOCP granules into a fine powder, which was then placed on a copper grid for observation. During the grinding process, a pestle and mortar were used to prevent any compositional changes while maintaining the particle size below 10 μm.

### Ex vivo model

**Fig. 2:**
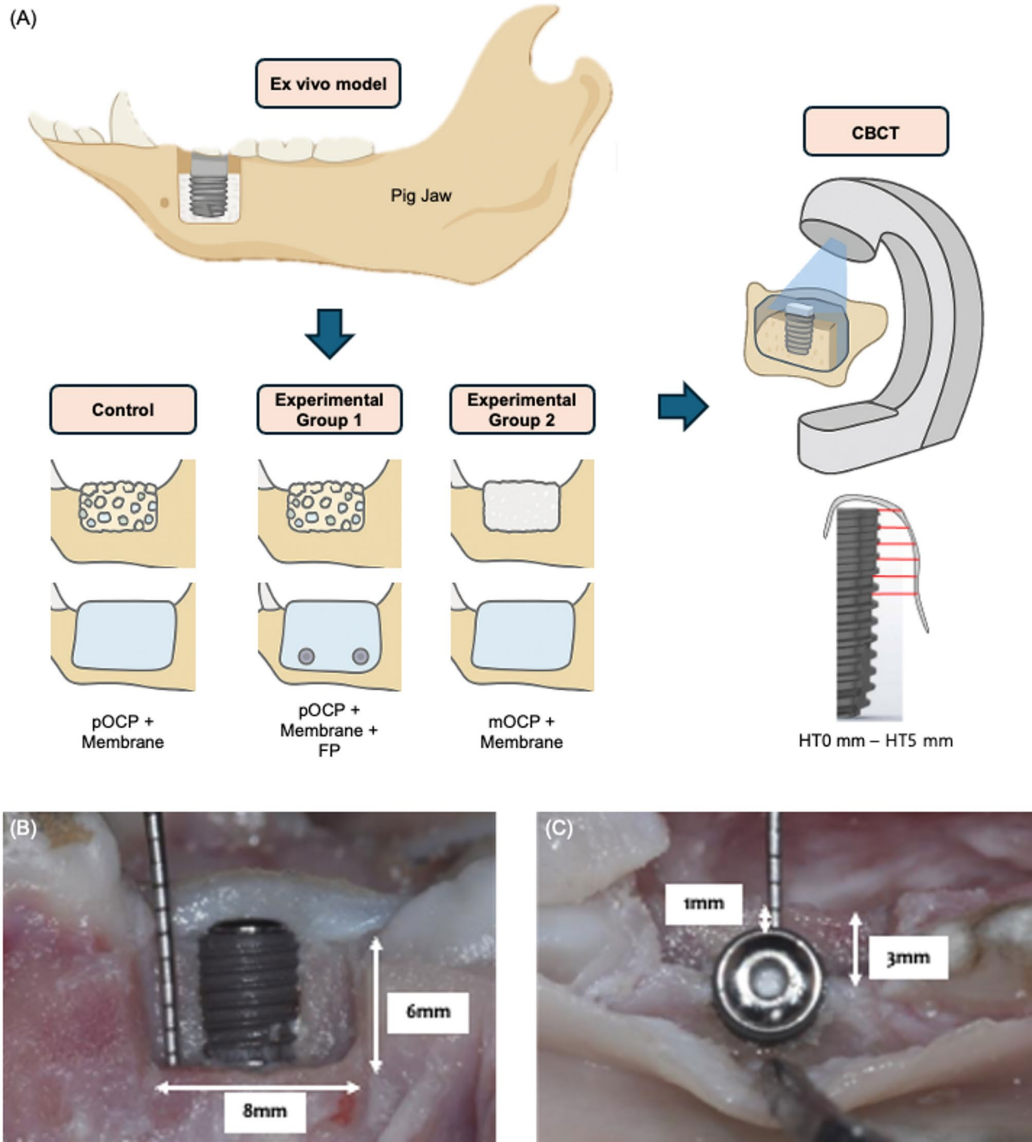
Schematic diagram of the ex vivo experimental setup and experimental peri-implant bone defect. (**A**) Schematic illustration of the experimental design including the three study groups and CBCT measurement approach. (**B**) Buccal and (**C**) occlusal views of the experimental peri-implant bone defect. pOCP; particulate octacalcium phosphate, FP; fixation pin, mOCP; moldable octacalcium phosphate. HT X mm; horizontal thickness of the augmented region measured X mm apical to the implant shoulder

In this study, bone defects were created following the ex vivo methodology established by Mir-Mari et al. [27] using pig mandibles. Bone defects were induced on both sides of the mandibles from ten pigs aged 5 to 6 months, one day after their sacrifice. A crestal incision was made, commencing distally from the second premolar and accompanied by a vertical releasing incision mesial to the second premolar. Subsequently, a total of 60 standardized box-shaped defects were generated—20 defects per group—between the second premolar and the first molar using a cylindrical carbide drill. The configuration of the bone defect was as follows: 8 mm from mesial to distal, 3 mm from buccal to lingual, and 6 mm apical to the alveolar crest. The objective of this study was to reproduce a clinical situation in which GBR was performed simultaneously with implant placement. A box-shaped bone defect was created in a porcine mandible to simulate a vertical and horizontal alveolar bone loss in the form of an intraosseous defect or an intraosseous defect immediately after tooth extraction [28]. An implant measuring 4.0 mm in diameter and 10.0 mm in height (JUST IMPLANT^®^, KJ Meditech, Gwangju, Korea) was then inserted into each defect, with the central axis aligned along the lingual wall and equidistant from both the mesial and distal walls. The shoulder of the implant was placed at the most coronal part of the lingual wall. Additionally, the distance between the buccal surface of the implant and the buccal edge of the defect, measured perpendicularly to the implant’s central axis, was maintained at 1 mm (Fig. 2). This experimental design was based on the standardized ex vivo model proposed by Mir-Mari et al. [27], allowing for methodological consistency and meaningful comparison with previous GBR studies.

### Experimental design

**Fig. 3:**
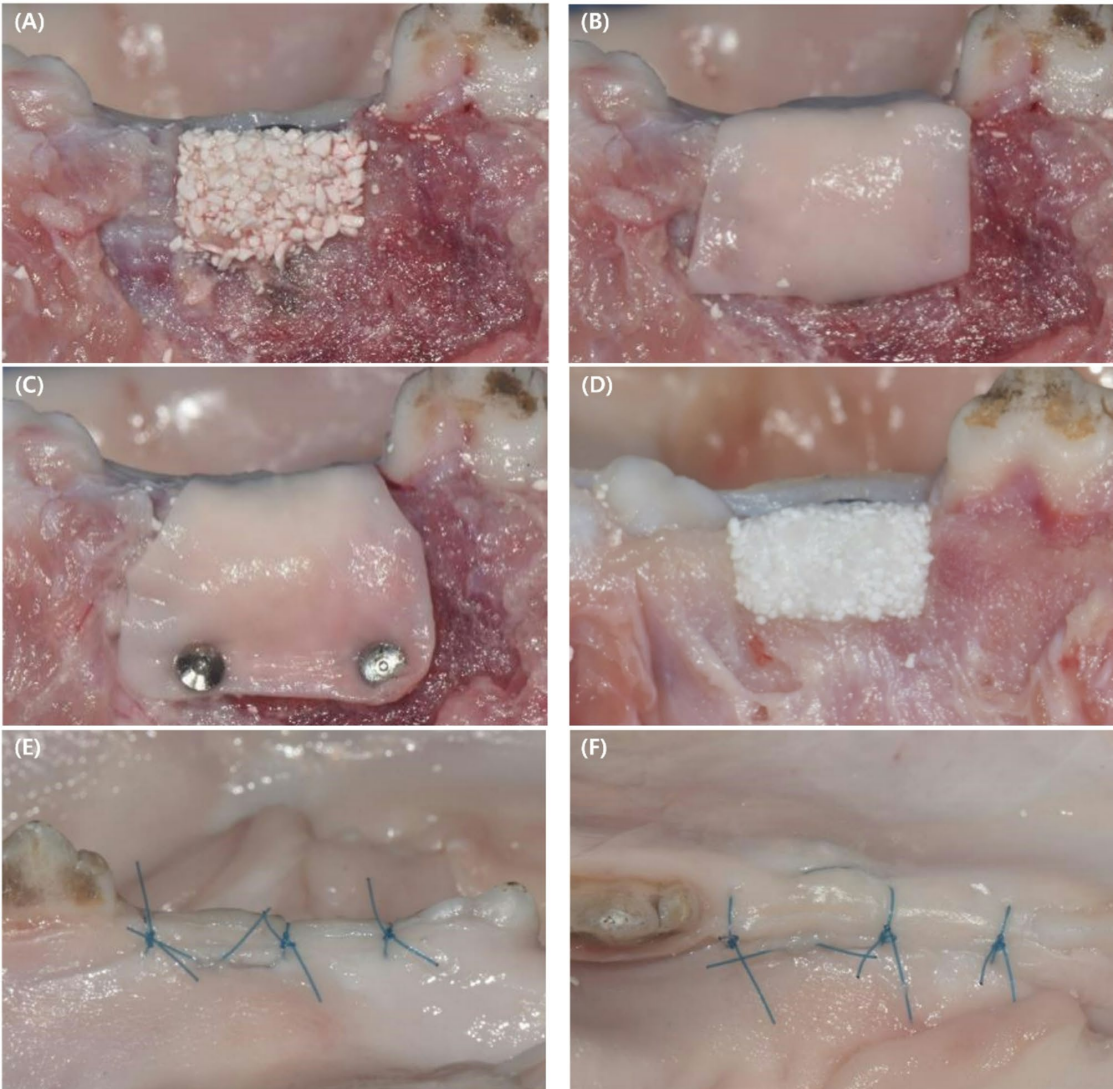
Surgical procedure. (**A**) A particulate octacalcium phosphate (OCP) bone substitute was grafted onto the buccal aspect of the defect. (**B**) A collagen membrane was then applied and covered. (**C**) The collagen membrane was covered, and the collagen barrier was fixed with fixation pins. (**D**) A moldable OCP bone substitute was grafted onto the buccal aspect of the defect. (**E**) The buccal view after suturing is shown. (**F**) The occlusal view after suturing is shown

Prior to bone grafting, the bone graft material and resorbable membrane were both treated with a radiopaque contrast medium to enhance visibility under CBCT. Specifically, 77% Ultravist 370 (Bayer, Zurich, Switzerland) was diluted in physiological saline at a 4:1 ratio (saline to contrast medium). The bone graft materials were gently mixed with the contrast solution to ensure homogeneous distribution, while the resorbable membrane was briefly immersed in the same solution prior to application. This preparation was performed immediately before placement to maintain consistency across all experimental groups. A total of 60 bone defects were assigned equally into three groups for testing (Fig. 3).

1) Control group: pOCP + resorbable membrane (*n* = 20).

2) Experimental Group I: pOCP + resorbable membrane + FPs (*n* = 20).

3) Experimental Group II: mOCP + resorbable membrane (*n* = 20).

### Experimental procedure

Following the creation of a box-shaped defect in the ex vivo pig mandible, the implant was placed. The bone graft material was added to the alveolar bone defect with the aim of achieving an overcontour of approximately 2 mm from the buccal surface. A resorbable membrane was then positioned over the bone graft, extending 2 mm beyond the edges of the defect. When using bone graft material and FP, two pins were inserted 1 to 2 mm apically from the lower edge of the apical wall of the defect to secure the resorbable membrane. For primary closure, a periosteal releasing incision was made at the base of the buccal flap to allow approximately 5 mm of coronal advancement, and simple interrupted sutures were applied to the crestal and vertical incisions using polyamide suture (Rexlon 5 − 0; SM Eng, Busan, Korea).

### Radiological evaluation

**Fig. 4:**
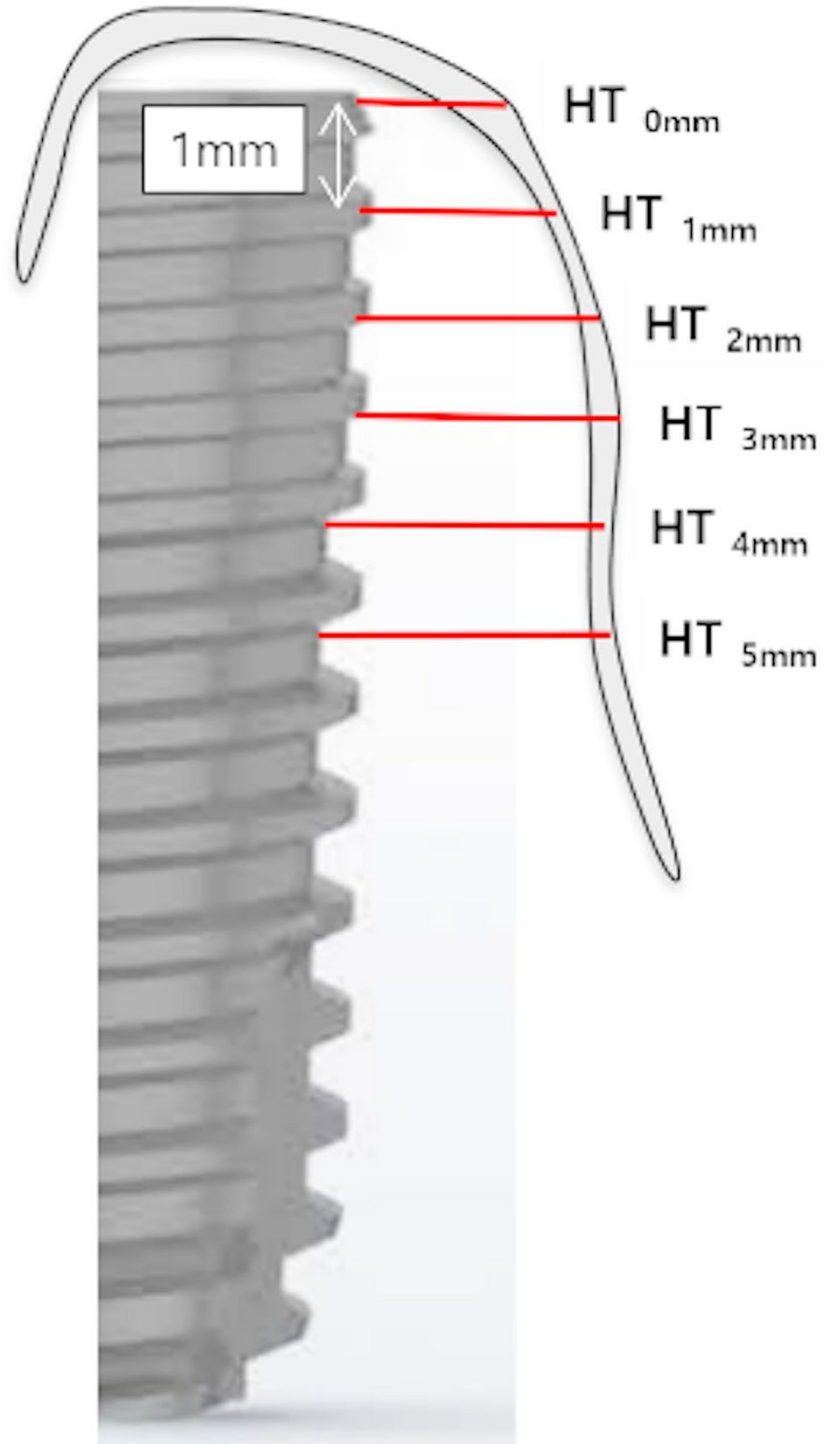
Schematic diagram of an implant to the implant central axis. Schematic diagram of the implant shoulder (HT0 mm) at intervals of 1, 2, 3, 4, and 5 mm (HT1 mm- HT5 mm) perpendicular to the implant central axis

CBCT scans (Planmeca Viso G7; Planmeca, Helsinki, Finland) were performed on the mandible immediately prior to and following flap closure at each site. During the radiographic procedure, the mandible was positioned in a support device, ensuring that the occlusal plane was parallel to a horizontal plane and centered within the field of view (FOV) using a laser-guided beam. The CBCT scans were performed with the following settings: acceleration voltage of 120 kVp, beam current of 40 mA, a field of view measuring 17 cm x 17 cm, and a voxel size of 0.15 mm. The analysis of CBCT DICOM data was conducted using the OnDemand 3D application (Ondemand, London, United Kingdom). The horizontal thickness of the bone graft was measured at intervals of 1, 2, 3, 4, and 5 mm (HT1 mm to HT5 mm) from the implant shoulder (HT0 mm) to the apical end [31, 32]. To standardize measurements and minimize operator bias, all radiological measurements were performed by a single calibrated examiner. The CBCT images acquired before and after suturing were saved as DICOM files, and measurements were repeated twice by the same examiner at a one-week interval to assess intra-examiner reliability. The mean value of the two measurements was used for analysis. The intra-operator reliability was verified using intraclass correlation coefficients (ICC), all exceeding 0.90. These measurements were obtained from cross-sectional images taken perpendicular to the central axis of the implant (Fig. 4).

### Data analysis

The change in horizontal thickness (HT) was evaluated both in absolute terms (mm) and relative terms (%), based on the differences in radiologically measured values before and after flap suturing. The statistical analyses were conducted using the SPSS 27.0 software (SPSS; IBM, Armonk, NY, USA). The descriptive statistics were calculated for all parameters, and the data distributions for the continuous variables are presented as bar graphs and box plots. The experimental values obtained from each group were subjected to analysis using mean, standard deviation (SD), and 95% confidence intervals (CI). The assumption of normality was evaluated through the application of the Kolmogorov-Smirnov and Shapiro-Wilk tests, with all HT changes demonstrating compliance with the requisite normality criteria. Inferential statistics were conducted using repeated measures ANOVA and paired samples t-tests. A repeated-measures ANOVA was utilized to assess the presence of inconsistencies in HT values across the three parameters. The paired samples t-test was applied to determine significant differences in HT changes for each method of bone augmentation before and after suturing, provided that normality was confirmed. The Wilcoxon test was utilized when normality was not achieved. This approach was consistently applied to analyze HT values before and after suturing based on the bone augmentation method.

## Results

### Characterization of mOCP

#### Phase analysis of bone graft materials

**Fig. 5:**
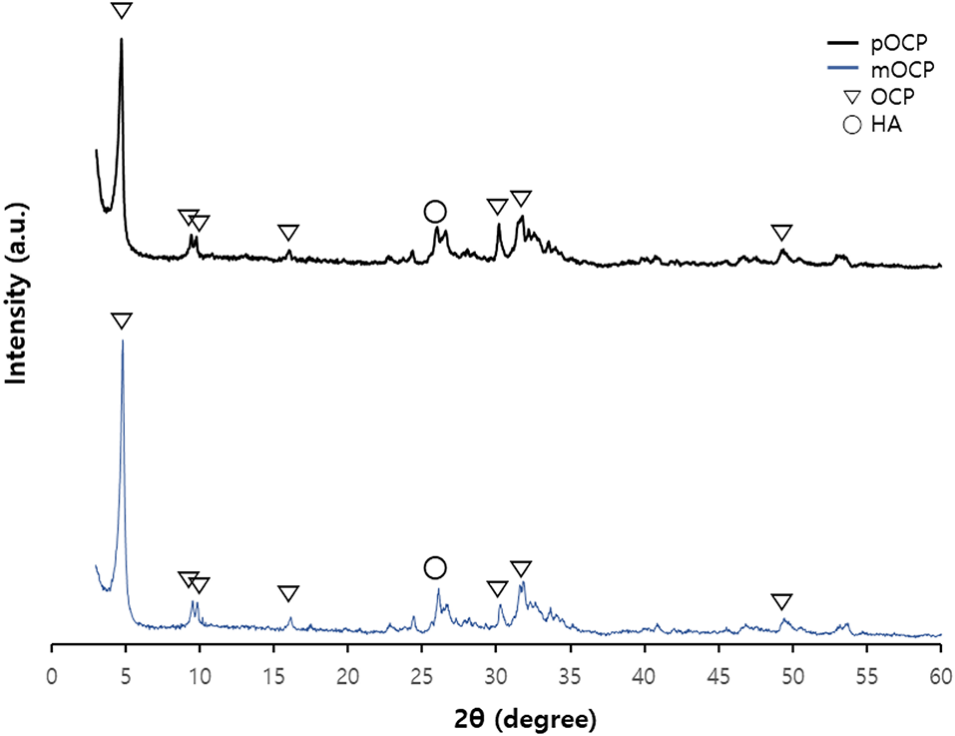
X-ray diffraction patterns of particulate OCP (pOCP) and moldable OCP (mOCP). OCP; octacalcium phosphate, HA; hydroxyapatite

As illustrated in Fig. 5, the XRD patterns of pOCP and mOCP exhibit comparable characteristics, suggesting that both samples align well with the defining peaks of OCP upon indexing. Although hydrogels typically generate a notable level of background noise in XRD analysis, the mOCP in this study showed minimal noise due to the reduced amount and thin coating of hydrogel, resulting in noise levels comparable to those of pOCP.

### Morphology of mOCP

**Fig. 6:**
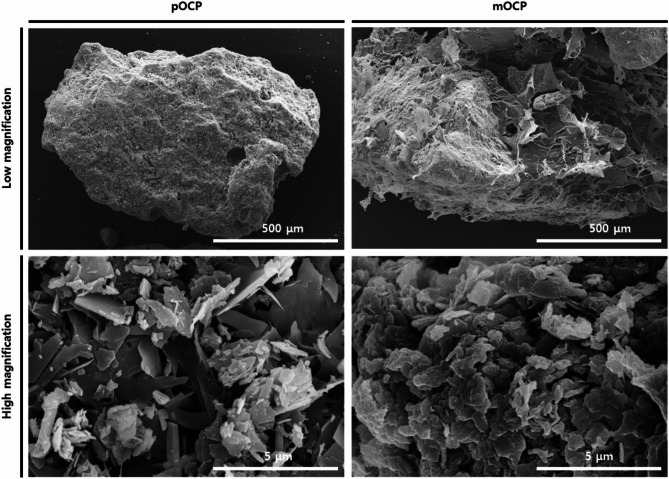
Scanning electron microscopy images of pOCP and mOCP. pOCP; particulate octacalcium phosphate, mOCP; moldable octacalcium phosphate

Scanning electron microscopy (SEM) analyses were conducted on the morphologies of pOCP and mOCP at low (100×) and high (10,000×) magnifications. The SEM images demonstrated that a significant portion of the graft surface comprising mOCP was coated with hydrogel (Fig. 6). Additionally, it was observed that the hydrogel was applied without any adverse effects on the porosity of the graft surface. Consequently, mOCP demonstrates moldable properties.

### Porosity measurement

The porosity was calculated from the amount of mercury that had intruded under pressure and was found to be 71.1143%. The characteristics of the nanoporosity are summarized in Table 1. When the porosity exceeds 70%, the samples demonstrate a proclivity to react with surrounding moisture rapidity, resulting in a viscous texture.

**Table 1:** Pore characteristics of moldable octacalcium phosphate

Total intrusion volume	3.397 ml/g	Average pore diameter (4 V/A)	978.51 nm
Total pore area	13.887 m^2^/g	Bulk density	0.2093 g/ml
Median pore volume	332,651.19 nm	Apparent (skeletal) density	0.7247 g/ml
Median pore area	153.77 nm	Porosity	71.1143%

### TEM-EDS results of mOCP

**Fig. 7:**
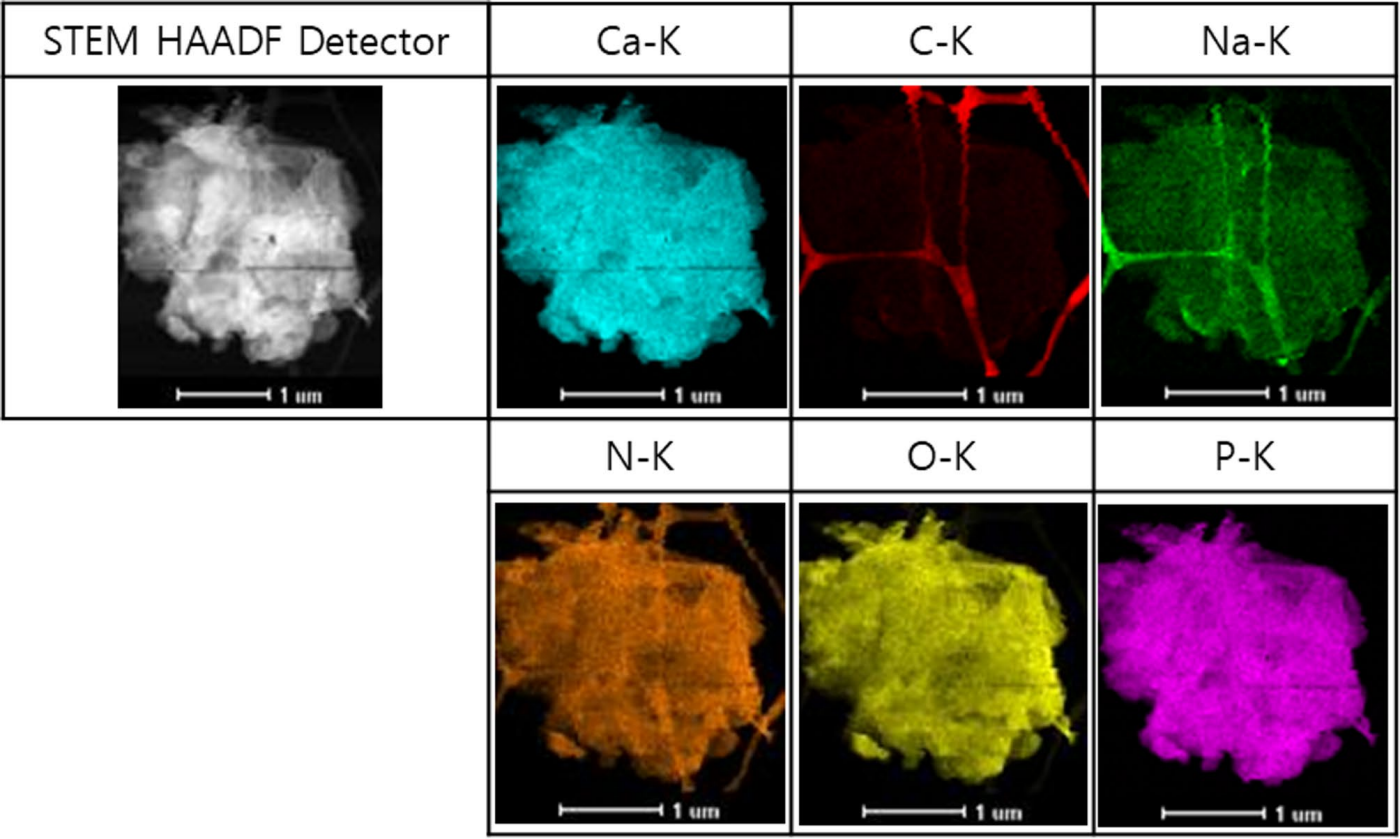
Transmission electron microscopy-energy dispersive spectrometry images of moldable octacalcium phosphate

The analysis of mOCP using TEM-EDS demonstrated a uniform distribution of essential elements throughout the sample. The elements calcium (Ca), phosphorus (P), oxygen (O), and hydrogen (H) were identified in the OCP structure, as were carbon (C) and sodium (Na) from sodium hyaluronate (Fig. 7). The concentrations of all elements were quantified and are presented in Table 2. Among these elements, oxygen constituted the highest proportion, followed by calcium, phosphorus, and carbon. The Ca/P ratio was calculated based on the proportions of calcium and phosphorus alone, resulting in a ratio of 1.28 for mOCP. This value is comparable to the theoretical Ca/P ratio of 1.33 for OCP.

**Table 2:** Elemental content of moldable octacalcium phosphate using transmission electron microscopy-energy dispersive spectrometry

Element	Atomic (%)
C	10.27
N	1.45
O	52.41
Na	3.47
P	14.20
Ca	18.16
Total	100

### Ex vivo model

**Fig. 8:**
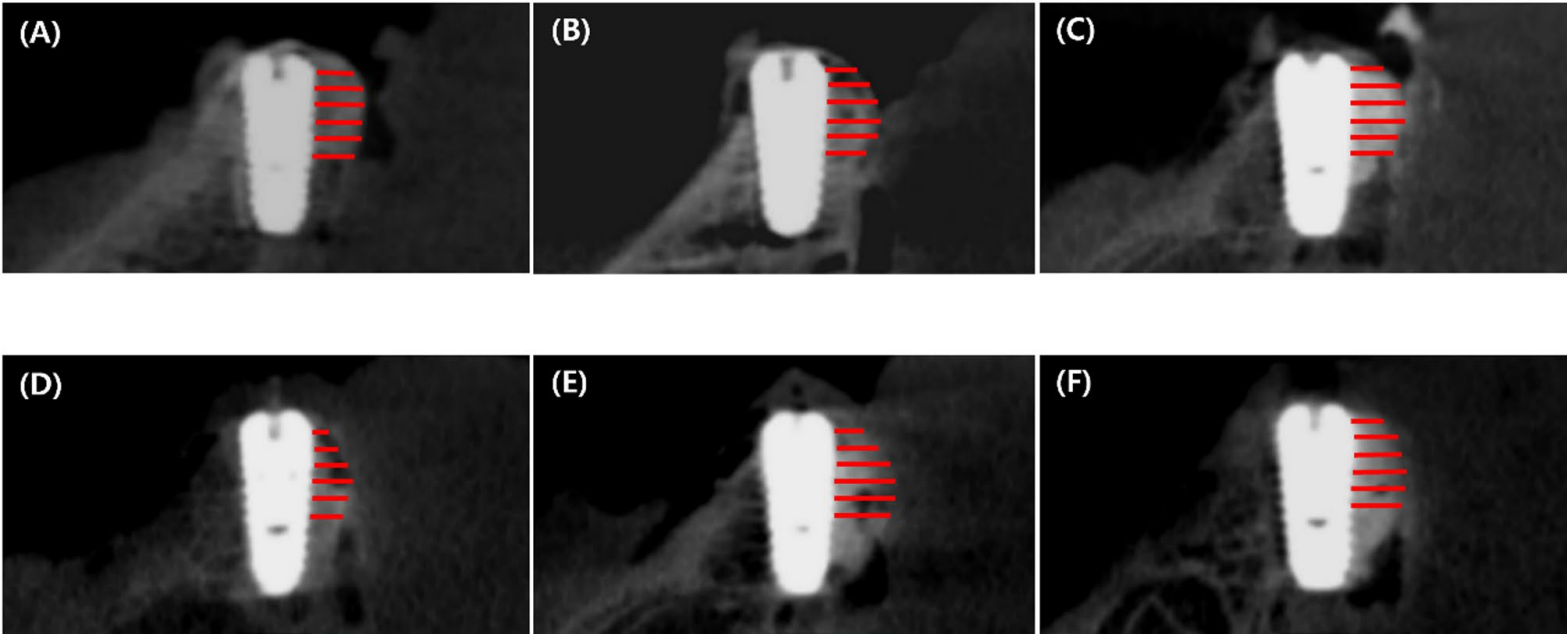
Cone Beam Computed Tomography (CBCT) reconstructions images. Bucco-oral CBCT reconstructions with the measurements of the dimensions of the augmented regions (HT0 mm–HT5 mm). (**A**),(**B**),(**C**): before suturing, (**D**),(**E**),(**F**): after suturing; (**A**),(**D**): Control group (pOCP + CM), (**B**),(**E**): Experimental group I (pOCP + CM + FP), and (**C**),(**F**): Experimental group II (mOCP + CM). pOCP; particulate octacalcium phosphate, CM; collagen membrane, FP; fixation pin, mOCP; moldable octacalcium phosphate

**Fig. 9:**
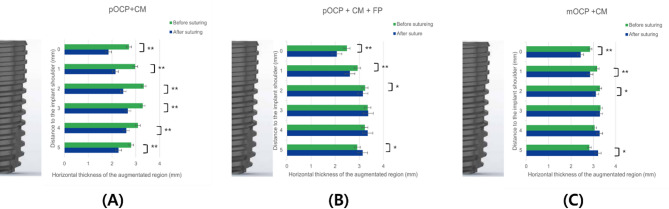
The bar plots of the horizontal thicknesses of the augmented regions before and after suturing. The bar plots illustrate the horizontal thicknesses of the augmented regions at various apico-coronal levels (HT0 mm–HT5 mm) before and after suturing for the following groups: (**A**) Control group (pOCP + CM), (**B**) Experimental group I (pOCP + CM + FP), and (**C)** Experimental group II (mOCP + CM). * *P* < 0.05 difference of horizontal thickness between before suture and after suture, ** *P* < 0.001 difference of horizontal thickness between before suture and after suture. pOCP; particulate octacalcium phosphate, CM; collagen membrane, FP; fixation pin, mOCP; moldable octacalcium phosphate

**Fig. 10:**
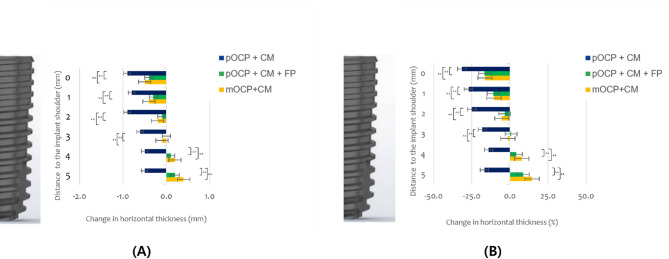
The bar plots of the changes in horizontal thickness of the augmented regions during the suturing procedures. The bar plots illustrate the changes in horizontal thickness of the augmented regions during the suturing procedures for the Control group (pOCP + CM), Experimental group I (pOCP + CM + FP), and Experimental group II (mOCP + CM). The data are presented in millimeters (**A**) and as a percentage (**B**). * *P* < 0.05 difference of change of horizontal thickness between this group and other group, ** *P* < 0.001 difference of change of horizontal thickness between this group and other group. pOCP; particulate octacalcium phosphate, CM; collagen membrane, FP; fixation pin, mOCP; moldable octacalcium phosphate

The radiographic measurements before and after suturing, HT results, and changes in HT according to the GBR process are presented in Tables 3 and 4; Figs. 8 and 9, and 10. The change in HT0 mm was measured as 31.5 ± 11.4% for the control group, 16.7 ± 8.5% for experimental group I, and 16.3 ± 11.7% for experimental group II. The change in HT1 mm was measured as 27.1 ± 12.4% for the control group, 11.0 ± 7.9% for experimental group I, and 10.4 ± 11.3% for experimental group II. The change in HT2 mm was measured as 25.0 ± 11.7% for the control group, 3.4 ± 7.8% for experimental group I, and 5.3 ± 11.4% for experimental group II. There was a statistically significant difference in the changes in HT0 mm, HT1 mm, and HT2 mm among the groups (control group HT0–2 mm, *P* < 0.001; experimental group I HT0 ~ 1 mm, *P* < 0.001, experimental group I HT2 mm, *P* = 0.038; experimental group II HT0–1 mm, *P* < 0.001, experimental group II HT2 mm, *P* = 0.031). The changes in HT3 mm and HT4 mm showed statistically significant results only in the control group (Control group HT3 mm, *P* < 0.001). The changes in HT5 mm showed statistically significant differences in all groups (Control group HT5 mm, *P* = 0.005; Experimental group I HT5 mm, *P* = 0.032; Experimental group II HT5 mm, *P* = 0.022) (Table 3; Fig. 9). The changes in HT0–5 mm between the control group and experimental group I (HT0–5 mm, *P* < 0.001) and the changes in HT0–5 mm between the control group and experimental group II (HT0.1 mm, *P* = 0.002; HT3–5 mm, *P* < 0.001) showed statistically significant differences. The changes in HT0–5 mm between experimental groups I and II did not show statistically significant differences in all areas (HT0 mm, *P* = 0.672; HT1 mm, *P* = 0.860; HT2 mm, *P* = 0.561; HT3 mm, *P* = 0.330; HT4 mm, *P* = 0.561; HT5 mm, *P* = 0.239) (Table 4; Fig. 10).

**Table 3:** The results of the horizontal thickness of the augmented region (HT) and the change in HT at different apico-coronal levels for control group (pOCP + CM), experimental group I (pOCP + CM + FP), experimental group II (mOCP + CM)

Treatment modality	Parameter	Before suturing (mm)	After suturing (mm)	Change (mm)	Change (%)	*P* value*
		Mean ± SD [95% CI]				
Control group (*n* = 20)	HT_0 mm_	2.7 ± 0.5	1.8 ± 0.4	-0.9 ± 0.4	-31.5 ± 11.4	*p* < 0.001
	HT_1 mm_	3.0 ± 0.5	2.1 ± 0.4	-0.8 ± 0.5	-27.1 ± 12.4	*p* < 0.001
	HT_2 mm_	3.3 ± 0.5	2.5 ± 0.4	-0.9 ± 0.5	-25.0 ± 11.7	*p* < 0.001
	HT_3 mm_	3.3 ± 0.6	2.7 ± 0.5	-0.6 ± 0.5	-18.1 ± 13.2	*p* < 0.001
	HT_4 mm_	3.1 ± 0.6	2.6 ± 0.5	-0.5 ± 0.6	-13.9 ± 17.1	*p* = 0.001
	HT_5 mm_	2.8 ± 0.7	2.3 ± 0.7	-0.5 ± 0.8	-16.7 ± 21.6	*p* = 0.005
Experimental group I (*n* = 20)	HT_0 mm_	2.6 ± 0.5	2.1 ± 0.4	-0.4 ± 0.2	-16.7 ± 8.5	*p* < 0.001
	HT_1 mm_	2.8 ± 0.4	2.6 ± 0.4	-0.3 ± 0.2	-11.0 ± 7.9	*p* < 0.001
	HT_2 mm_	3.0 ± 0.4	3.1 ± 0.5	-0.1 ± 0.2	-3.4 ± 7.8	*p* = 0.038
	HT_3 mm_	3.0 ± 0.4	3.4 ± 0.5	0.0 ± 0.2	0.8 ± 7.1	*p* = 0.631
	HT_4 mm_	2.9 ± 0.5	3.4 ± 0.5	0.1 ± 0.3	4.2 ± 11.0	*p* = 0.092
	HT_5 mm_	2.6 ± 0.5	3.2 ± 0.5	0.2 ± 0.4	8.7 ± 17.0	*p* = 0.032
Experimental group II (*n* = 20)	HT_0 mm_	2.8 ± 0.7	2.4 ± 0.6	-0.5 ± 0.4	-16.3 ± 11.7	*p* < 0.001
	HT_1 mm_	3.2 ± 0.6	2.8 ± 0.6	-0.4 ± 0.4	-10.4 ± 11.3	*p* = 0.001
	HT_2 mm_	3.3 ± 0.6	3.1 ± 0.6	-0.2 ± 0.4	-5.3 ± 11.4	*p* = 0.031
	HT_3 mm_	3.3 ± 0.6	3.3 ± 0.6	-0.1 ± 0.3	-1.3 ± 10.6	*p* = 0.414
	HT_4 mm_	3.1 ± 0.7	3.3 ± 0.7	0.2 ± 0.5	7.8 ± 16.9	*p* = 0.069
	HT_5 mm_	2.8 ± 0.8	3.2 ± 0.9	0.4 ± 0.6	14.5 ± 27.2	*p* = 0.022

n; number, HT X mm; horizontal thickness of the augmented region measured X mm apical to the implant shoulder, SD; standard deviation, 95% CI; 95% confidence interval

*; results of repeated-measures ANOVA with Greenhouse–Geisser correction

pOCP; particulate octacalcium phosphate, CM; collagen membrane, FP; fixation pin, mOCP; moldable octacalcium phosphate

**Table 4:** The following section presents the results of the change in horizontal thickness of the augmented region for the various treatment procedures for control group (C; pOCP + CM), experimental group I (E1; pOCP + CM + FP), experimental group II (E2; mOCP + CM)

	Change (mm)				Change (%)			Statistical analysis*	
	Control	E1	E2		Control	E1	E2		
	Mean ± SD [95% CI]								
HT_0 mm_	-0.9 ± 0.4	-0.4 ± 0.2	-0.5 ± 0.4		-31.5 ± 11.4	-16.7 ± 8.5	-16.3 ± 11.7	C vs. E1	< 0.001
								C vs. E2	0.002
								E1 vs. E2	0.672
HT_1 mm_	-0.8 ± 0.5	-0.3 ± 0.2	-0.4 ± 0.4		-27.1 ± 12.4	-11.0 ± 7.9	-10.4 ± 11.3	C vs. E1	< 0.001
								C vs. E2	0.002
								E1 vs. E2	0.860
HT_2 mm_	-0.9 ± 0.5	-0.1 ± 0.2	-0.2 ± 0.4		-25.0 ± 11.7	-3.4 ± 7.8	-5.3 ± 11.4	C vs. E1	< 0.001
								C vs. E2	< 0.001
								E1 vs. E2	0.561
HT_3 mm_	-0.6 ± 0.5	0.0 ± 0.2	-0.1 ± 0.3		-18.1 ± 13.2	0.8 ± 7.1	-1.3 ± 10.6	C vs. E1	< 0.001
								C vs. E2	< 0.001
								E1 vs. E2	0.330
HT_4 mm_	-0.5 ± 0.6	0.1 ± 0.3	0.2 ± 0.5		-13.9 ± 17.1	4.2 ± 11.0	7.8 ± 16.9	C vs. E1	< 0.001
								C vs. E2	< 0.001
								E1 vs. E2	0.561
HT_5 mm_	-0.5 ± 0.8	0.2 ± 0.4	0.4 ± 0.6		-16.7 ± 21.6	8.7 ± 17.0	14.5 ± 27.2	C vs. E1	< 0.001
								C vs. E2	< 0.001
								E1 vs. E2	0.239

HT X mm; horizontal thickness of the augmented region measured X mm apical to the implant shoulder, SD; standard deviation, 95% CI; 95% confidence interval

*, results of repeated-measures ANOVA with Greenhouse–Geisser correction

pOCP; particulate octacalcium phosphate, CM; collagen membrane, FP; fixation pin, mOCP; moldable octacalcium phosphate

## Discussion

The objective of this study was to investigate the displacement patterns of bone graft materials before and after suturing, taking into account the presence or absence of membrane fixation and the type of bone graft material. To this end, an ex experimental model with pig mandibles was employed. In all groups, a notable amount of collapse of the augmented bone graft material was observed in the coronal region, which is consistent with the findings reported by Mir-Mari et al. [27]. In contrast, the supplementary administration of FP in experimental group I and the utilization of mOCP bone substitutes in experimental group II demonstrated a notable reduction in the unavoidable elevation in bone graft failure following suturing.

It is common for the incorporation of hydrogels to result in a considerable amount of noise in the XRD peaks. However, in this study, the mOCP exhibited minimal noise due to the reduced amount of hydrogel used and its thin coating, resulting in a similarity in noise levels compared to the XRD peaks of pOCP. The SEM images show that a significant portion of the graft surface composed of mOCP is coated with hydrogel, but the thin coating of hydrogel seems to be applied without negatively affecting the porosity of the graft surface. The results of these experiments show that mOCP exhibits moldable properties. In general, when the porosity exceeds 70%, the sample tends to react quickly with the surrounding moisture, resulting in a sticky texture. In this study, mOCP showed a porosity of 71.1143%, which appears to be responsible for its sticky characteristics. The mOCP bone substitute, as utilized in this study, displays a viscosity comparable to that of clay when combined with water. This property may help maintain graft placement during bone augmentation, provide adaptability to various defect types, and support mechanical stability [12]. The Ca/P ratio of mOCP was calculated based only on the ratio of calcium and phosphorus, and the ratio of mOCP was 1.28. This value is similar to the theoretical Ca/P ratio of OCP, which is 1.33. Therefore, it can be seen that mOCP still has similar characteristics to conventional OCP. In contrast to biphasic calcium phosphate (BCP), a composite of HA and β-tricalcium phosphate that typically exhibits slow degradation and primarily functions as a passive space-maintaining scaffold [33–35], OCP has been recognized for its superior bioactivity owing to its transitional conversion to hydroxyapatite under physiological conditions. This transformation plays a key role in directly stimulating osteoblastic differentiation and accelerating new bone formation [9–11]. While BCP tends to remain partially unresorbed and contributes mainly to mechanical support, OCP actively participates in biological remodeling. The mOCP used in this study retains these bioactive properties and demonstrated stable application performance during experimental use.

Hydrogels are characterized by their physical and chemical properties, which are similar to human tissues. They have controllable degradability, biocompatibility, and good mechanical properties, which allows them to have a wide range of action prospects. In general, the properties of hydrogels improve with increasing concentration, which means that the biocompatibility and degradation of hydrogels are low. This contradiction limits the application of hydrogels in the medical field. Most hydrogels exhibit favorable adhesion in dry environments, but in humid or underwater environments, the hydrogel surface swells and water molecules form, which can significantly reduce or even loss adhesion [18]. However, it is noteworthy that in this study, only a small amount of thin coating is required to achieve the advantages of improved manipulability and moldable application even in moist hemorrhage sites. It is also noteworthy that the use of hydrogels, as compared to previous PRFs or CGFs, can exhibit these properties.

It is well documented that collagen membranes produce favorable clinical outcomes [4, 36, 37]. However, the limited stiffness of resorbable collagen membranes reduces their capacity to maintain space, which can result in partial or complete collapse of the barrier [30, 38]. Consequently, resorbable membranes may not facilitate the same degree of volume gain over time as non-resorbable membranes [39]. This limitation can also be attributed to their temporary barrier function [30, 40]. It can be concluded that resorbable membranes require supplementary reinforcement from the underlying bone substitute material for optimal stabilization [19]. In recent years, there has been an increasing preference for performing GBR at the same time as implant placement, rather than the staged method of placing implants after GBR. This shift can be attributed to the duration of treatment and the number of visits required. The objective of this study was to create a model that would allow for the reproduction of a clinical situation in which GBR was performed in a simultaneous manner with implant placement. This was achieved by creating a box-shaped bone defect in a porcine mandible, simulating the case of immediate post-extraction implant placement or the intraosseous type of bone loss [28]. In this study, flap suturing after GBR in peri-implant bone defects induced significant displacement of bone graft thickness, and the displacement was most pronounced at the implant shoulder level. These results showed that although tension-free sutures were used, the compressive force on the increased area during suturing could not be completely avoided. However, moldable OCP showed stable performance in the implant shoulder area even without the use of FP.

Several approaches have been proposed for the purpose of reducing the displacement of graft materials. For example, the utilization of vertical mattress sutures to secure absorbable membranes has been demonstrated to augment the stability of particulate bone grafts [26]. Nevertheless, this approach is not without limitations, primarily due to the tensile strength of the sutures, which provides fixation only during the biodegradation period of the absorbable sutures. A reduction in tensile strength has been observed in these sutures, with a decrease to 50–60% of their initial strength occurring within one week after insertion and a further reduction to 20–30% after two weeks. Consequently, the sutures may be absorbed before sufficient revascularization and biological bone remodeling can occur in the graft material. Furthermore, the tensile force exerted by the sutures securing the membrane may result in the collapse of the particulate bone graft material at the implant shoulder region [41–43]. As an alternative approach, the stabilization of the resorbable membrane with FP has been proposed. The additional support provided can effectively reduce the displacement of the bone substitute material during the process of wound closure [27, 44]. Previous research indicates that GBR using a resorbable membrane and FP resulted in approximately a 30% reduction in changes compared to cases without fixation [44]. The use of FP has been shown to effectively prevent and stabilize the collapse of both the bone graft material and the membrane [45]. Additionally, stabilizing the proximal end of the resorbable membrane with a pin can decrease the degree of scattering or displacement of the particulate bone graft material during suturing from about 40–20% [27].

Experimental groups I and II demonstrated greater stability in horizontal thickness changes before and after suturing compared to the control group, with values of 16.7% for Experimental group I and 16.3% for Experimental group II. Significant differences were observed between the control group and each experimental group. However, no significant differences were observed between the two experimental groups. The findings indicate that the utilization of FP and the implementation of an mOCP substitute are both efficacious in maintaining the stability of the bone substitute, even in the post-suturing phase of bone augmentation. Furthermore, the mOCP substitute showed stability comparable to that achieved with FP in the implant shoulder region, even in the absence of FP. When compared to earlier studies, our findings reveal similar trends regarding the use of particulate bone substitutes and resorbable membranes, as well as the application of FP with resorbable membranes [27]. In the previous study [27], the control group without fixation experienced a horizontal decrease of 42.8% at HT0 mm, while the combination of particulate bone graft material and FP resulted in a decrease of 22.9% at HT0 mm. In our study, the control group exhibited a decrease of 31.5% at HT0 mm. The use of resorbable membranes with FP led to a decrease of 16.7%, while the mOCP substitute showed a decrease of 16.3%. These results align with previous literature and indicate that the mOCP substitute could serve as a stable alternative to FP in bone grafting applications.

The observed reduction in volume in the implant shoulder region, coupled with the expansion in the apical area, can be ascribed to the compression and displacement effects of the bone graft material. The findings demonstrated that the control group exhibited a horizontal reduction in the apical region without a corresponding increase in the graft material, indicating a loss of the graft material. However, both experimental group I and experimental group II demonstrated a slight increase in the apical region as a consequence of the displacement effect. In particular, the mOCP bone substitute showed an increase in volume at the apical end, which can be explained by better adhesion between the bone particles. This appears to be related to the increased viscosity of mOCP mentioned above. It appears to cluster in the apical region instead of falling out due to the increased resistance to washout by body fluids.

The stability of GBR can be enhanced by the effective fixation of the barrier membrane and bone graft material. A fundamental element of effective GBR is the reduction of micro-movement of the graft material, as any displacement can potentially result in complications such as soft tissue dehiscence or inadequate blood clot formation. In light of these findings, previous research has proposed the utilization of FP in the context of extensive bone grafting procedures. Nevertheless, the utilization of these pins may entail certain disadvantages, including the potential for damage to the anatomical structures of adjacent roots and the necessity for additional surgical intervention to remove the pins, which could complicate their use [26, 29, 46]. It can be reasonably inferred that mOCP has the potential to be utilized as a reliable bone graft material, as it provides comparable stability to FP while eliminating the risk of root injury and the additional surgical step required for pin removal. This characteristic may contribute to the simplification of GBR procedures in clinical practice by reducing operative time, minimizing postoperative discomfort, and enhancing overall treatment efficiency.

However, it should be noted that this study has the limitation of representing an experiment that only partially simulates the clinical situation of GBR for peri-implant defects. It is possible that ex vivo conditions do not accurately replicate in vivo clinical environments, as they do not facilitate physiological processes such as blood clot formation, vascularization, and soft tissue integration. This may affect the adhesive and cohesive properties of the particulate bone graft, potentially resulting in greater collapse or displacement of the graft material. The experimental setting also posed specific technical challenges, including the dehydration of the porcine skin flap and the drying out of the contrast agent mixed with the graft material, which reduced the maneuverability of the graft and affected defect shape maintenance. In addition, the use of CBCT imaging in an ex vivo model has inherent limitations in assessing clinical outcomes. Radiological images are susceptible to distortion due to artifacts and metal interference, which can hinder the accurate detection of small bone defects or peri-implant radiolucency [47, 48]. Although three-dimensional analysis using CBCT allowed for volumetric assessment in this study, further research employing histological analysis and micro-CT imaging could help validate these radiographic findings. Furthermore, the present study does not provide conclusions regarding the long-term stability or biological behavior of the augmented ridge. Although the mechanical stability of the graft material was evaluated, factors such as biodegradation rate and biological integration can significantly influence clinical outcomes. Previous histological studies on collagen-based equine bone blocks have reported significant biodegradation over time, which may result in altered ridge contour and soft tissue volume [49, 50]. Future studies using in vivo animal models or clinical trials in humans are therefore necessary to confirm the biological performance of mOCP under physiological healing conditions, including blood clot retention, inflammatory response, and remodeling dynamics. Finally, it is important to acknowledge the relatively small sample size of this study. Although no formal sample size calculation was performed, the number of defects per group was determined based on previously established ex vivo GBR models, particularly the study by Mir-Mari et al. [27], to ensure methodological consistency and comparative validity. Prospective in vivo studies with larger sample sizes and longer follow-up periods are warranted to fully validate the clinical applicability and long-term efficacy of mOCP in bone regeneration procedures.

## Conclusions

In light of the constraints inherent to this study, the use of mOCP bone substitutes demonstrated favorable outcomes comparable to the FP approach in terms of volumetric stability. Moreover, by eliminating the need for fixation pins, mOCP may contribute to simplification of surgical procedures. Consequently, the application of mOCP in bone augmentation may could be a feasible option to support predictability and efficiency of GBR procedures.

## Data Availability

No datasets were generated or analysed during the current study.
